# Mesophotic.org: a repository for scientific information on mesophotic ecosystems

**DOI:** 10.1093/database/baz140

**Published:** 2019-12-14

**Authors:** Pim Bongaerts, Gonzalo Perez-Rosales, Veronica Z Radice, Gal Eyal, Andrea Gori, Erika Gress, Nicholas M Hammerman, Alejandra Hernandez-Agreda, Jack Laverick, Paul Muir, Hudson Pinheiro, Richard L Pyle, Luiz Rocha, Joseph A Turner, Ryan Booker

**Affiliations:** 1 California Academy of Sciences, San Francisco, CA 94118, USA; 2 Global Change Institute, The University of Queensland, St Lucia, QLD 4072, Australia; 3 PSL Research University: EPHE-UPVD-CNRS, USR 3278 CRIOBE, Université de Perpignan, Perpignan 66860, France; 4 ARC Centre of Excellence for Coral Reef Studies, The University of Queensland, St Lucia, QLD 4072, Australia; 5 The Mina & Everard Goodman Faculty of Life Sciences, Bar Ilan University, Ramat Gan 5290002, Israel; 6 Dipartimento di Scienze e Tecnologie Biologiche e Ambientali, Università del Salento, Lecce 73100, Italy; 7 Nekton Foundation, Oxford, OX5 1PF, UK; 8 Department of Zoology, University of Oxford, OX1 3SZ, UK; 9 Queensland Museum, Townsville, QLD 4810, Australia; 10 Bernice P. Bishop Museum, Honolulu, HI 96817, USA; 11 Ocean Ecology Ltd, Severnside Park, Epney, GL2 7LN, UK; 12 Global Underwater Explorers, High Springs, FL 32643, USA; 13 Departament de Biologia Evolutiva, Ecologia i Ciències Ambientals, Universitat de Barcelona, Barcelona 08028, Spain

## Abstract

Mesophotic coral ecosystems (MCEs) and temperate mesophotic ecosystems (TMEs) occur at depths of roughly 30–150 m depth and are characterized by the presence of photosynthetic organisms despite reduced light availability. Exploration of these ecosystems dates back several decades, but our knowledge remained extremely limited until about a decade ago, when a renewed interest resulted in the establishment of a rapidly growing research community. Here, we present the ‘mesophotic.org’ database, a comprehensive and curated repository of scientific literature on mesophotic ecosystems. Through both manually curated and automatically extracted metadata, the repository facilitates rapid retrieval of available information about particular topics (e.g. taxa or geographic regions), exploration of spatial/temporal trends in research and identification of knowledge gaps. The repository can be queried to comprehensively obtain available data to address large-scale questions and guide future research directions. Overall, the ‘mesophotic.org’ repository provides an independent and open-source platform for the ever-growing research community working on MCEs and TMEs to collate and expedite our understanding of the occurrence, composition and functioning of these ecosystems.

Database URL: http://mesophotic.org/

## Background & Summary

Mesophotic coral ecosystems (MCEs) occur in the deeper parts of the ocean’s photic zone, beyond the limits of regular scientific diving but shallow enough to still support photosynthetically-active organisms. Traditionally, the term ‘mesophotic’ (literally ‘middle-light’) was occasionally used in the scientific literature in referral to aquatic depth layers of low-light (1–3). However, the first published mention in reference to extant coral reef ecosystems was by Robert Ginsburg at a meeting of the Association of Marine Laboratories of the Caribbean in 2007 (4). The term was later officially adopted during an international workshop organized by the U.S. National Oceanic and Atmospheric Administration (NOAA) in 2008 (5), primarily to distinguish deeper sections of (sub)tropical coral reefs from entirely non-photosynthetic deep-sea and cold-water coral ecosystems (6). According to the NOAA definition, ‘mesophotic coral ecosystems (MCEs) are characterized by the presence of light-dependent corals and associated communities typically found at depths ranging from 30 to 40 m and extending to over 150 m in tropical and subtropical regions’ (5, 7). Following that, the term ‘mesophotic’ started to be more broadly used to refer to benthic habitats within the depth zone of 30–150 m depth, including in temperate waters (8, 9) where communities at those depths are now characterized as ‘temperate mesophotic ecosystems’ (10).

**Figure 1:**
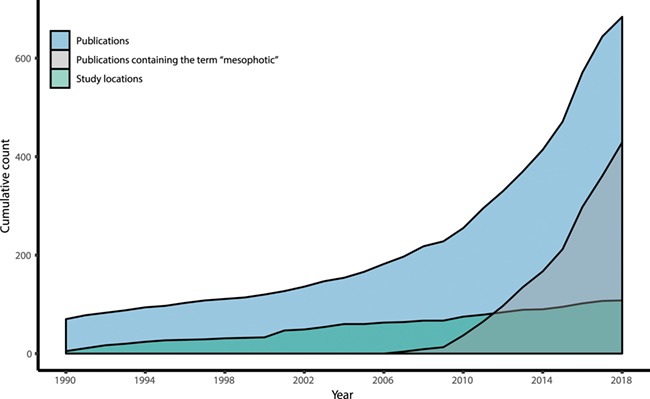
The quantity and geographic spread of scientific literature on mesophotic ecosystems is increasing over time. Series show the cumulative count of scientific publications on mesophotic ecosystems, the subset of those publications using the term ‘mesophotic’, and unique research locations in the current ‘mesophotic.org’ database release.

Exploration of MCEs began in the 1960s and 1970s by manned submersibles (e.g. 11, 12) and early pioneers of deep scuba diving (13). However, research efforts on MCEs remained extremely limited until its establishment as a ‘field of research’ about a decade ago (5, 7). The renewed interest in MCEs has been driven by their potential connection with shallow-water coral reef communities as potential ‘refuges’ from major disturbances (14, 15), their unique and often undescribed biodiversity (6, 16), and their increased accessibility due to the advancement of underwater technologies (e.g. mixed-gas scuba, closed-circuit rebreathers and remotely operated vehicles). Overall, this has led to a major increase in scientific attention over the past decade (17), as evidenced by the rapid growth in the quantity and geographic spread of published scientific literature (Figure 1).

Considering the rapid decline of shallow reefs due to the effects of climate change (18, 19), MCEs have become a common interest among not only the scientific community but also governmental organizations and non-profit groups (10, 20). There is an emerging consensus that despite their potential role as refuges, MCEs are not immune to disturbance and instead are vulnerable in their own right (16, 21, 22). In order to consider appropriate management for MCEs, it is critical to assess them under different pressures (23). While remaining largely undocumented, MCEs are estimated to occupy similar or larger areas of habitat compared to shallow coral reefs (6, 24, 25), with most of this habitat not included in conservation management efforts (26–28). In order to collate the scientific literature and provide a platform for the growing research community, a basic web repository was launched at the ‘International Workshop to Prioritize Research and Management Needs for Mesophotic Coral Ecosystems’ organized by NOAA in 2008 (5). The repository has since progressed to form the current iteration of ‘mesophotic.org’ (http://mesophotic.org): a comprehensive and curated repository of scientific literature on MCEs and TMEs (hereafter referred to as ‘mesophotic ecosystems’). The main goals of this repository are to (i) provide a comprehensive and dynamic record of all the published scientific information on these ecosystems and make it queryable through a single web portal, (ii) allow for the exploration of spatial/temporal trends and identification of knowledge gaps through curated metadata for each publication and (iii) act as an institution-independent platform and as a shared resource for and by the research community to accelerate our understanding regarding the occurrence, composition and functioning of these ecosystems.

## Database information and usage

The current release provides a comprehensive collection of peer-reviewed, scientific articles up to the end of 2018 and incorporates efforts from several other systematic literature reviews (6, 10, 29). The primary contents of ‘mesophotic.org’ are therefore currently peer-reviewed publications, although the proportion of secondary scientific literature including conference proceedings, technical reports, museum publications, book chapters and (post)graduate theses is growing (as we continue to add these). Each catalogued publication is first identified as ‘mesophotic’, depending on whether it presents data from or a discussion on the mesophotic depth zone (defined by the most commonly considered depth range of 30–150 m depth; 30) or whether it is relevant despite not occurring in this depth range. Next, where relevant, the publication is tagged either as ‘mesophotic coral ecosystem’ or ‘temperate mesophotic ecosystem’ to facilitate separate searches and summaries for these distinct systems. The publications are identified by type (scientific, technical and popular), format (article, review, report, chapter, book and thesis) and whether they present original data and/or report new species.

In addition to standard citation information, additional metadata are manually extracted from each publication (an up-to-date overview of fields and categories can be found on http://mesophotic.org/metadata), including the minimum and maximum study depth, the geographic location(s) (each defined by a latitude and longitude), the research platform(s) (e.g. ‘rebreather’), the research field(s) (e.g. ‘physiology’) and the focal taxa (e.g. ‘Scleractinia’). The abstract and full text are automatically indexed to extract the occurrence (frequency) of custom keywords (e.g. ‘mesophotic’), species known to occur at mesophotic depths (through a curated list: http://mesophotic.org/species) and metadata categories (as mentioned above). The article is also summarized through a ‘word cloud’ (eliminating undesired stop words through a custom ‘stop list’). This also allows keywords in the abstract and word cloud to be hyperlinked to other pages on the website. Full-text contents are stored in the database for indexing and searching purposes but are not accessible to comply with copyright regulations. An advanced search function is available to search across publications and their associated metadata, and the results can be browsed through the website or downloaded as a comma-separated values (CSV) file. If a search term is queried, publications are ordered by the number of occurrences of that term in the full text, which can be helpful in identifying the most relevant references. In addition, each metadata field has a separate entry page to summarize literature by category (e.g. geographic region, research platform or focus taxon).

New publications are added by the content editors but can be suggested (as well as any corrections) by any website visitor through email (info@mesophotic.org). After extracted metadata are entered, they are considered ‘validated’ when verified by at least two different content editors using an internal validation system. If a content editor changes any of the metadata, the system will expire existing validations prompting the other content editors for revalidation. In addition to scientific publications, the website maintains a moderated list of people working on mesophotic ecosystems, a gallery of member photos (with the option of sharing photos for reuse with the Creative Commons License; CC BY-NC 4.0) and a regular blog featuring scientists and their publications in ‘Behind the Science’ posts.

**Figure 2:**
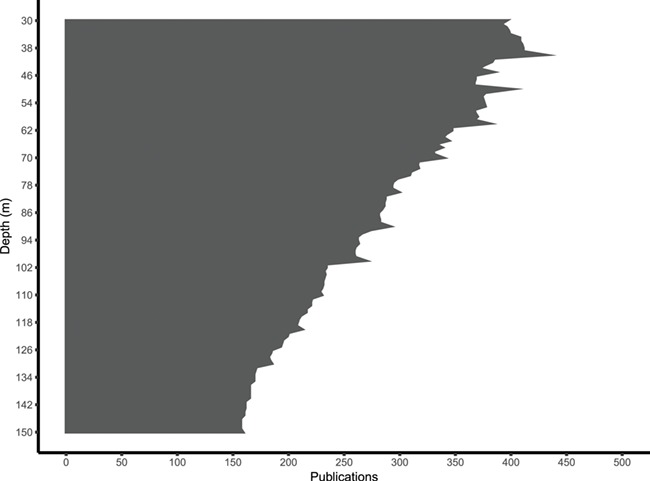
Number of scientific publications decreases over depth (30–150 m). The number of publications at each (1 m) depth interval is determined by assuming the entire depth range from minimum to maximum depth for each publication in the current release.

**Figure 3:**
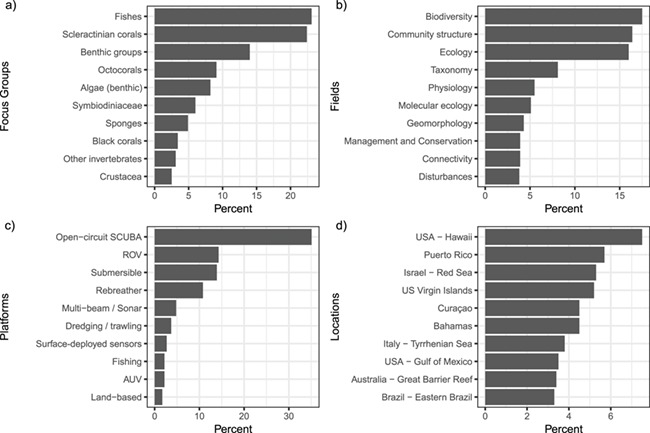
Top 10 fields of metadata categories of the curated literature on mesophotic ecosystems. a) 10 most-studied focus groups of mesophotic publications, b) 10 most-studied fields of research in mesophotic publications, c) 10 most common platforms used to conduct research at mesophotic depths and d) 10 locations with the greatest number of publications focused on mesophotic research.

**Figure 4:**
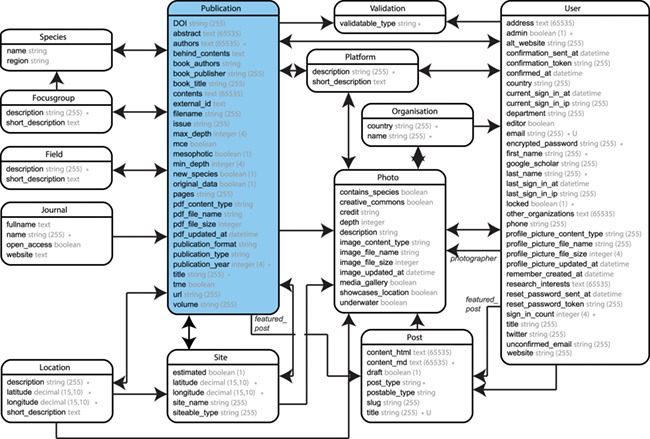
Entity Relationship Diagram (ERD) of the ‘mesophotic.org’ database release.

The current release of all the validated metadata associated with publications can be downloaded in CSV format from anywhere in the ‘mesophotic.org’ repository. Data can be directly imported into R for analysis by using the ‘read.csv’ option (www.r-project.org) and pointing to the URL of the dynamically generated CSV files on the website (e.g. http://mesophotic.org/publications.csv). Alternatively, CSV files of specific queries can be downloaded, saved and imported in the same way. An electronic notebook with usage examples, including reproductions of the figures included with this manuscript, can be found on http://mesophotic.org/tutorial. Metadata can also be explored through the Statistics page (http://www.mesophotic.org/stats), to provide general summaries of the data, such as the number of publications over time (Figure 1) and over depth (Figure 2) and the most commonly studied focal taxa, research fields, geographic locations or research platforms (Figure 3).

## Code Availability

The source code for ‘mesophotic.org’ was written in Ruby (2.5.0) on Rails (5.2.0) and is freely accessible through http://www.github.com/pimbongaerts/mesophotic. The source code is open-source and free to be used, shared and modified under the MIT License. The application relies on a wide range of Ruby gems and JavaScript packages that are detailed in the README file of the code repository. The database structure is visualized through an Entity Relationship Diagram (ERD; Figure 4), with the most recent version also accessible through the README file. Issues and bugs can be reported through the https://github.com/pimbongaerts/mesophotic/issues page.
